# Trends in hepatitis B virus seroprevalence in a large cohort of adult cancer patients from 2010 to 2023

**DOI:** 10.1007/s10552-026-02262-w

**Published:** 2026-10-07

**Authors:** Marina Rossi de Camargo Pinto, Victória Gonçalves de Paula, Débora Bignotto Rosane Battaglia, Rafael de Oliveira, Gustavo Manoel Ferreira, Caroline Manchiero, Bianca Peixoto Dantas, Fátima Mitiko Tengan, Edson Abdala, Mariana Cavalheiro Magri

**Affiliations:** 1 Laboratório de Investigação Médica em Hepatologia por Vírus (LIM-47), Hospital das Clínicas HCFMUSP, Faculdade de Medicina, Universidade de São Paulo, Sao Paulo (SP), Brazil; 2 Instituto de Medicina Tropical, Faculdade de Medicina, Universidade de São Paulo, Sao Paulo (SP), Brazil; 3 Departamento de Infectologia e Medicina Tropical, Faculdade de Medicina, Universidade de Sao Paulo, Sao Paulo (SP), Brazil; 4 Instituto do Câncer do Estado de São Paulo, Faculdade de Medicina, Universidade de São Paulo, Sao Paulo (SP), Brazil

**Keywords:** Cancer, Epidemiology, Hepatitis B virus, Seroprevalence, Temporal trends

## Abstract

**Purpose:**

Hepatitis B virus (HBV) infection poses increased risks for cancer patients due to immunosuppression and potential viral reactivation. We evaluated the seroprevalence and temporal trends of HBV exposure in a large cohort of cancer patients in Brazil.

**Methods:**

Adult patients who underwent anti-HBc testing at a quaternary oncology center between 2010 and 2023 were included in this retrospective study, and HBV exposure was defined as anti-HBc positivity. Temporal trends were assessed by using linear regression, whereas associated factors were evaluated by using multivariable logistic regression.

**Results:**

Anti-HBc testing was performed in 22.9% (28,869/125,985) of admitted cancer patients, and seroprevalence was 13.4% (3,857/28,869), declining from 16.5 in 2010 to 9.9% in 2023 (*p* < 0.001). Decreasing trends were observed across sexes and age groups (*p* < 0.001 for both), with a more pronounced decline among men (25.1%–12.3%). Seroprevalence also decreased significantly among White patients (16.7%–7.9%; *p* < 0.001) and those with solid tumors (18.8%–9.0%; p < 0.001), but not among non-White individuals (21.4%–17.7%; p = 0.246) or those with hematological malignancies (13.1%–12.8%; *p* = 0.136). Age ≥ 60 years (OR = 4.40; 95%CI: 3.77–5.12; *p* < 0.001), men (OR = 1.89; 95%CI: 1.76–2.04; *p* < 0.001), and non-White ethnicity/color (OR = 1.25; 95%CI: 1.15–1.35; *p* < 0.001) were independently associated with HBV exposure.

**Conclusion:**

Despite the declining seroprevalence over time, HBV exposure remains common among cancer patients, as persistent disparities across demographic groups underscore the need for expanded vaccination and systematic screening.

**Supplementary Information:**

The online version contains supplementary material available at https://doi.org/10.1007/s10552-026-02262-w.

## Introduction

Hepatitis B virus (HBV) infects hepatocytes and can establish a chronic infection, which may lead to severe complications, including liver cirrhosis and hepatocellular carcinoma [1]. HBV infection is recognized as one of the strongest risk factors for hepatocellular carcinoma, increasing the odds of disease development by approximately 47-fold [2]. Despite the availability of effective vaccination, an estimated 240 million individuals are living with chronic HBV infection, which is associated with substantial morbidity and mortality [3]. The clinical course of chronic HBV infection varies considerably among individuals, likely reflecting complex interactions between viral factors and host immune response. Moreover, viral reactivation may occur, particularly in the setting of immunosuppression [4, 5].

Systemic anticancer therapies can induce immunosuppression, facilitating viral replication and triggering HBV reactivation, even in patients with occult infection due to the persistence of covalently closed circular DNA (cccDNA) within the hepatocyte nucleus [6]. A recent global meta-analysis reported reactivation rates of approximately 10% among patients with hematological malignancies and 5% among those with solid tumors [7]. HBV reactivation may result in interruption, delay, or modification of anticancer therapy and may lead to acute hepatitis, liver failure, and death [8].

Patients with chronic HBV infection (HBsAg-positive) should receive antiviral therapy, in which entecavir, tenofovir disoproxil fumarate (TDF), or tenofovir alafenamide (TAF) are the preferred choices. Patients with HBsAg (hepatitis B surface antigen)-negative and anti-HBc (antibody against the hepatitis B core antigen)-positive results should be considered at risk for HBV reactivation, and strategies such as antiviral prophylaxis and/or serological and molecular monitoring should be implemented [4, 9]. Although international guidelines recommend HBV screening prior to initiating immunosuppressive therapy or chemotherapy [4, 9, 10], this practice is still inconsistently implemented across oncology centers [11, 12].

Epidemiological data on HBV exposure prevalence among patients with hematological malignancies and solid tumors are relevant for informing healthcare planning and management. Therefore, this study evaluated the seroprevalence and temporal trends of HBV exposure (anti-HBc positivity) in a large cohort of cancer patients from 2010 to 2023.

## Materials and methods

### Study setting and population

This retrospective observational study was conducted in the Laboratory of Viral Hepatology (*Laboratório de Investigação Medica em Hepatologia por Vírus*—LIM-47) of the Clinical Hospital, Faculty of Medicine, University of Sao Paulo (*Hospital das Clínicas, Faculdade de Medicina, Universidade de São Paulo*—HCFMUSP). The seroprevalence of HBV exposure was assessed by using laboratory data from the Sao Paulo State Cancer Institute (*Instituto do Câncer do Estado de São Paulo*—Icesp), Brazil, which is a specialized cancer center affiliated with the HCFMUSP. Since its inauguration in 2008, Icesp has treated more than 140,000 patients with cancer.

We included all patients with hematological malignancies or solid tumors who attended the Icesp between January 2010 and December 2023 and underwent anti-HBc testing during the study period. Patients younger than 18 years were excluded. All patients meeting these eligibility criteria over the 14-year study period were included to maximize the representativeness of the study population.

### Definitions

HBV exposure was defined as total anti-HBc seropositivity, which indicates previous exposure to the virus and can signify either a past or present infection.

### Data sources and collection

Serological data for anti-HBc were obtained from the institutional Central Laboratory Division (DLC-HCFMUSP), where all laboratory blood tests were performed. The variables extracted from the hospital’s electronic medical record system included demographic characteristics (i.e. age, sex, and ethnicity/color) and cancer diagnoses. Data were consolidated into a structured dataset, filtered, and cleaned by using Microsoft Power BI Desktop, version 2.145.1105.0 (Microsoft Corp., Redmond, WA, USA), before being statistically analysed.

Sex was defined according to sex assignment at birth (i.e. man or woman), and all individuals were aged ≥ 18 years. Ethnicity/color was self-reported and classified according to Brazilian census categories as follows: White (European-origin Brazilians), Black (African-origin Brazilians), Indigenous (Indigenous Brazilians), and Yellow (Asian-origin Brazilians). Individuals self-identifying as “Pardo” (Brown) were classified as mixed ethnicity.

Cancer diagnoses were retrieved from the institutional health information system (GIS-Icesp) and classified according to the International Classification of Diseases (ICD) codes for primary tumor site. Diagnoses were grouped as follows: (a) hematological malignancies encompassing Hodgkin lymphoma, leukemia, multiple myeloma, non-Hodgkin lymphoma, and other hematological malignancies; and (b) solid tumors encompassing bone, skin and soft tissue, breast, central nervous system, female genitourinary, gastrointestinal tract, head and neck, liver and bile ducts, male genitourinary, sarcoma, trachea, bronchus, and lung, and other solid tumors.

For patients who tested positive for anti-HBc, results from additional HBV serologic markers (HBsAg, anti-HBs, anti-HBc IgM, HBeAg, and anti-HBe) and HBV DNA testing performed during the same clinical evaluation were extracted from the institutional DLC database.

### Variables

Age was analysed both as a continuous and categorical variable (i.e. 18–39, 40–59, and ≥ 60 years). Sex was analysed as men *versus* women, and ethnicity/color as White *versus* non-White. Cancer types were categorized as hematological malignancies *versus* solid tumors. These variables were selected a priori based on their established epidemiological and clinical relevance to HBV infection and their potential role as determinants of anti-HBc positivity in patients with cancer.

### Statistical analysis

Descriptive statistics provided a summary of the study population’s characteristics. The proportion of anti-HBc tests performed was estimated as the number of anti-HBc tested cases divided by the total number of cancer patients admitted at Icesp. HBV seroprevalence was estimated as the number of anti-HBc positive cases divided by the total number of cancer patients who underwent testing for this serological marker, being expressed as proportions. Temporal trends were assessed according to the year of anti-HBc testing by using linear regression and then stratified by sex, age group, ethnicity/color, and cancer type. Associations of HBV exposure with cancer type and demographic variables were evaluated by using chi-square tests and logistic regression models, with results reported as odds ratios (ORs) and 95% confidence intervals (CIs). Variables with *p* < 0.20 in the bivariate analysis were included in the multivariable logistic regression model. A two-sided *p* < 0.05 was considered statistically significant. Analyses were performed by using the Statistical Package for Social Sciences (SPSS) software, version 22.0 (IBM Corp., Armonk, NY, USA).

## Results

### Patient characteristics

All adult cancer patients who underwent anti-HBc serological testing between 2010 and 2023 were included in the study (*n* = 28,869). The mean age of the study population was 56 ± 15.2 years, and the majority of the patients were women (65.4%; *n* = 18,888). Ethnicity/color was self-reported, with a predominance of White patients (61.4%; *n* = 17,721), followed by Pardo patients (10.7%; *n* = 3,088) (Table 1).

**Table 1:** Characteristics of 28,869 enrolled cancer patients, 2010–2023

Characteristic	Frequency
Age, years	
Mean ± SD	56 ± 15.2
Sex, *n* (%)	
Female	18,888 (65.4)
Male	9,981 (34.6)
Ethnicity/color, *n* (%)	
White	17,721 (61.4)
Black	1,669 (5.8)
Indigenous	54 (0.2)
Yellow	745 (2.6)
Pardo	3,088 (10.7)
Not reported	5,592 (19.3)
Cancer type, *n* (%)	
Hematological	6,406 (22.2)
Leukemia	1,142 (4.0)
Hodgkin lymphoma	968 (3.3)
Non-Hodgkin lymphoma	2,817 (9.8)
Multiple myeloma	1,175 (4.1)
Other—hematological	304 (1.0)
Solid	20,571 (71.2)
Bone, skin and soft tissue	751 (2.6)
Breast	6,726 (23.3)
Central nervous system	281 (1.0)
Female genitourinary	4,654 (16.1)
Gastrointestinal tract	3,276 (11.3)
Head and neck	1,193 (4.1)
Liver and bile ducts	804 (2.8)
Male genitourinary	1,864 (6.5)
Sarcoma	23 (0.1)
Trachea, bronchus and lung	504 (1.7)
Other—solid	495 (1.7)
Missing	1,892 (6.6)

*SD* standard deviation

As for cancer type, 22.2% (*n* = 6,406) of the patients had hematological malignancies and 71.2% (*n* = 20,571) had solid tumors. Among hematological malignancies, non-Hodgkin lymphoma (9.8%; *n* = 2,817) and multiple myeloma (4.1%; *n* = 1,175) were the most frequent subgroups. Among solid tumors, breast cancer (23.3%; *n* = 6,726) and female genitourinary cancers (16.1%; *n* = 4,654) predominated (Table 1).

In comparative analyses (*n* = 26,977), patients with hematological malignancies were younger and more frequently men, whereas those with solid tumors were more often women and aged ≥ 60 years (*p* < 0.001 for both). White patients predominated in both groups, with a higher proportion observed among those with hematological malignancies (*p* < 0.001) (Table 2).

**Table 2:** Characteristics of patients, distributed among hematological malignancies and solid tumors, 2010–2023

Variables	Hematological (*n* = 6,406)	Solid (*n* = 20,571)	*p*
Age, years	–	–	< 0.001
18–39	1,537 (24.0)	2,665 (13.0)	–
40–59	2,145 (33.5)	8,434 (41.0)	–
≥ 60	2,724 (42.5)	9,472 (46.0)	–
Sex	–	–	< 0.001
Female	2,938 (45.9)	14,368 (69.8)	–
Male	3,468 (54.1)	6,203 (30.2)	–
Ethnicity**/**color^a^	–	–	< 0.001
White	4,331 (67.6)	12,084 (58.7)	–
Non-White	1,163 (18.2)	4,063 (19.8)	–

Data expressed as *n* (%), Chi-square test

^a^No data: 5,336

### Seroprevalence and temporal trends of HBV exposure

**Fig. 1:**
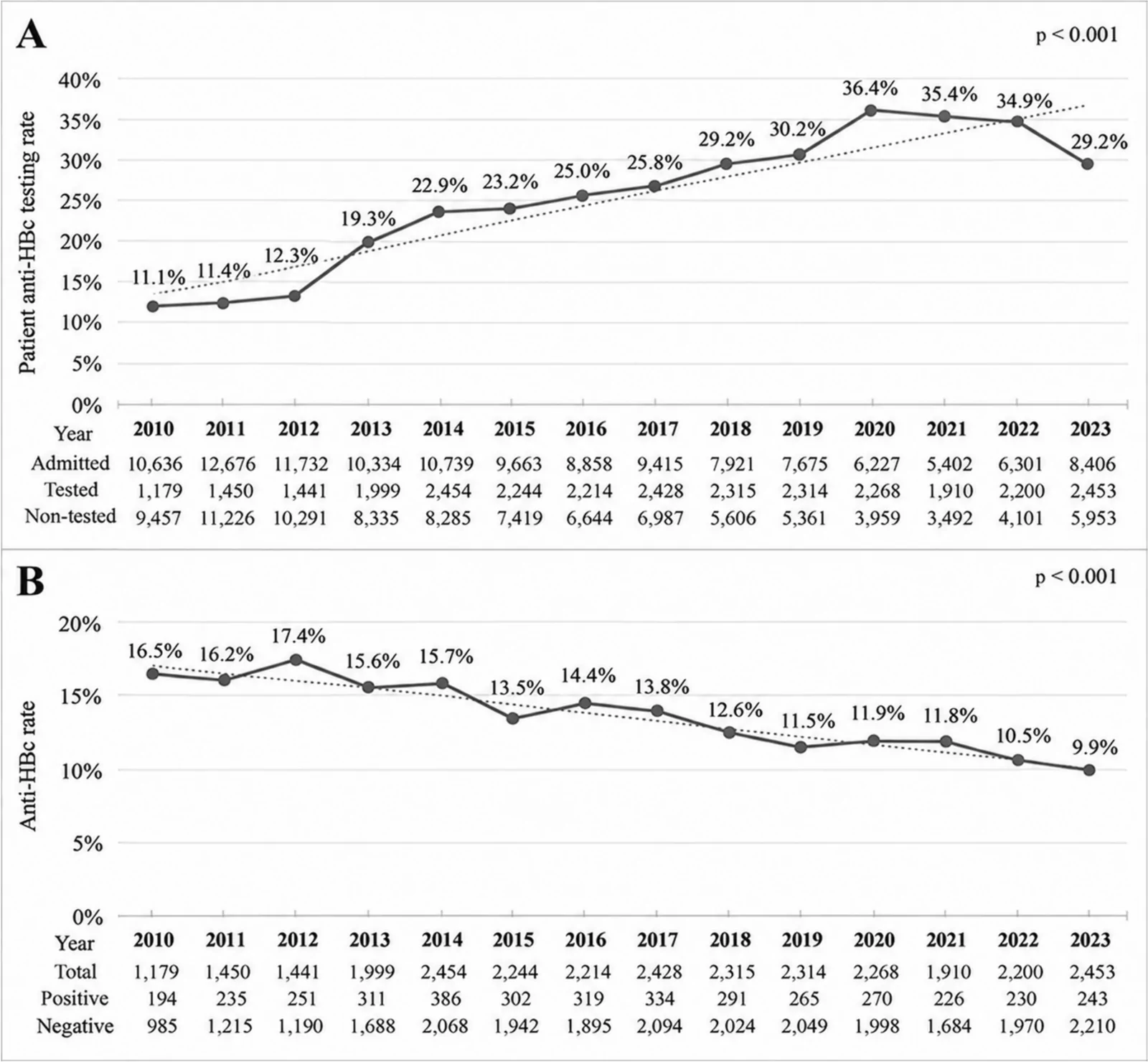
Trends in the proportion of cancer patients **a** tested for anti-HBc and **b** positive for anti-HBc (hepatitis B virus exposure) by year of study, 2010–2023. *Anti-HBc* antibody against the hepatitis B core antigen

A total of 125,985 patients were admitted at Icesp during the study period, of whom 22.9% (*n* = 28,869) underwent anti-HBc testing. A significant upward trend in the proportion of tested patients was observed over the 14-year study period (*p* < 0.001), increasing from 11.1% (1,179/10,636) in 2010 to 29.2% (2,453/8,406) in 2023 (Fig. 1A).

The overall seroprevalence of HBV exposure among cancer patients was 13.4% (3,857/28,869). A significant downward trend was observed over the 14-year study period (*p* < 0.001), with seroprevalence decreasing from 16.5% (194/1,179) in 2010 to 9.9% (243/2,453) in 2023 (Fig. 1B). The annual number of patients undergoing anti-HBc testing increased over the study period, from 1,179 in 2010 to 2,453 in 2023 (Fig. 1B).

**Fig. 2:**
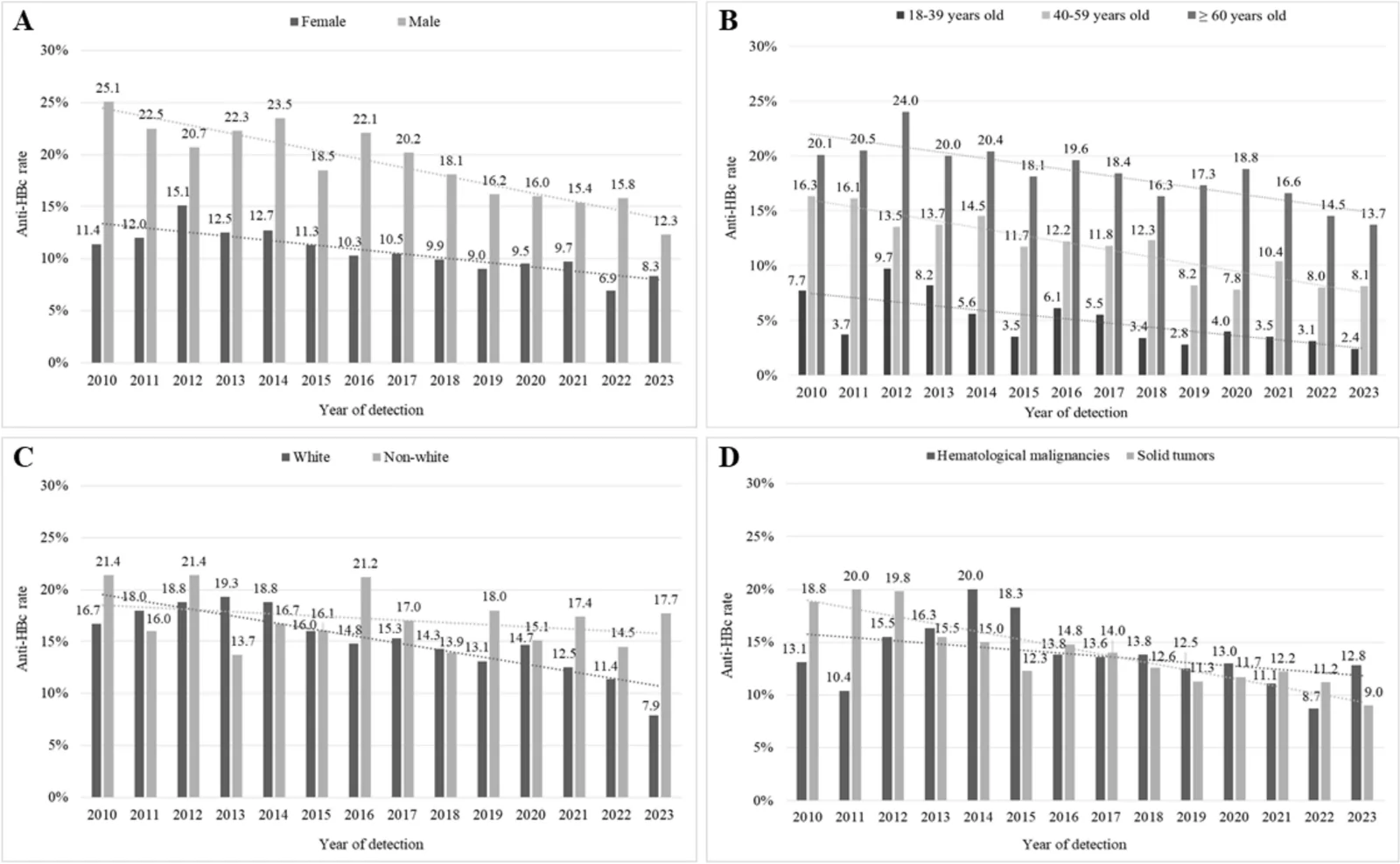
Changes in the annual seroprevalence of hepatitis B virus exposure among cancer patients, according to **a** sex, **b** age group, **c** ethnicity/color, and **d** cancer type, 2010–2023. *Anti-HBc* antibody against the hepatitis B core antigen

Temporal trends stratified by sex, age, ethnicity/color, and cancer type are presented in Fig. 2. The seroprevalence of HBV exposure decreased significantly over time (2010 to 2023) across most subgroups. Downward trends were observed among both women (11.4% to 8.3%; *p* < 0.001) and men (25.1% to 12.3%; *p* < 0.001). Likewise, all age groups showed a decline, including patients aged 18–39 years (7.7% to 2.4%; *p* = 0.004), 40–59 years (16.3% to 8.1%; *p* < 0.001), and ≥ 60 years (20.1% to 13.7%; *p* < 0.001). A significant decline was also observed among White patients (16.7% to 7.9%; *p* < 0.001) and those with solid tumors (18.8% to 9.0%; *p* < 0.001), whereas no significant trend was observed among non-White individuals (21.4% to 17.7%; *p* = 0.246) or patients with hematological malignancies (13.1% to 12.8%; *p* = 0.136).

### HBV serological and molecular profile among anti-HBc-positive patients

Among the 3,857 anti-HBc-positive patients, additional HBV serologic and molecular markers were evaluated in a subset of individuals, although not all tests were performed for everyone. HBsAg was positive in 299/3,840 (7.8%) patients, anti-HBs in 2,808/3,835 (73.2%), anti-HBc IgM in 18/564 (3.2%), HBeAg in 58/558 (10.4%), and anti-HBe in 209/284 (73.6%). HBV DNA testing was performed for 484 patients, of whom 124 (25.6%) had detectable viral load (Supplementary Table S1). Overall, these findings indicate that the majority of anti-HBc-positive patients had serological evidence consistent with past HBV exposure, while a smaller subset presented with serological and/or molecular evidence suggestive of current HBV infection.

### Seroprevalence of HBV exposure according to cancer type

**Fig. 3:**
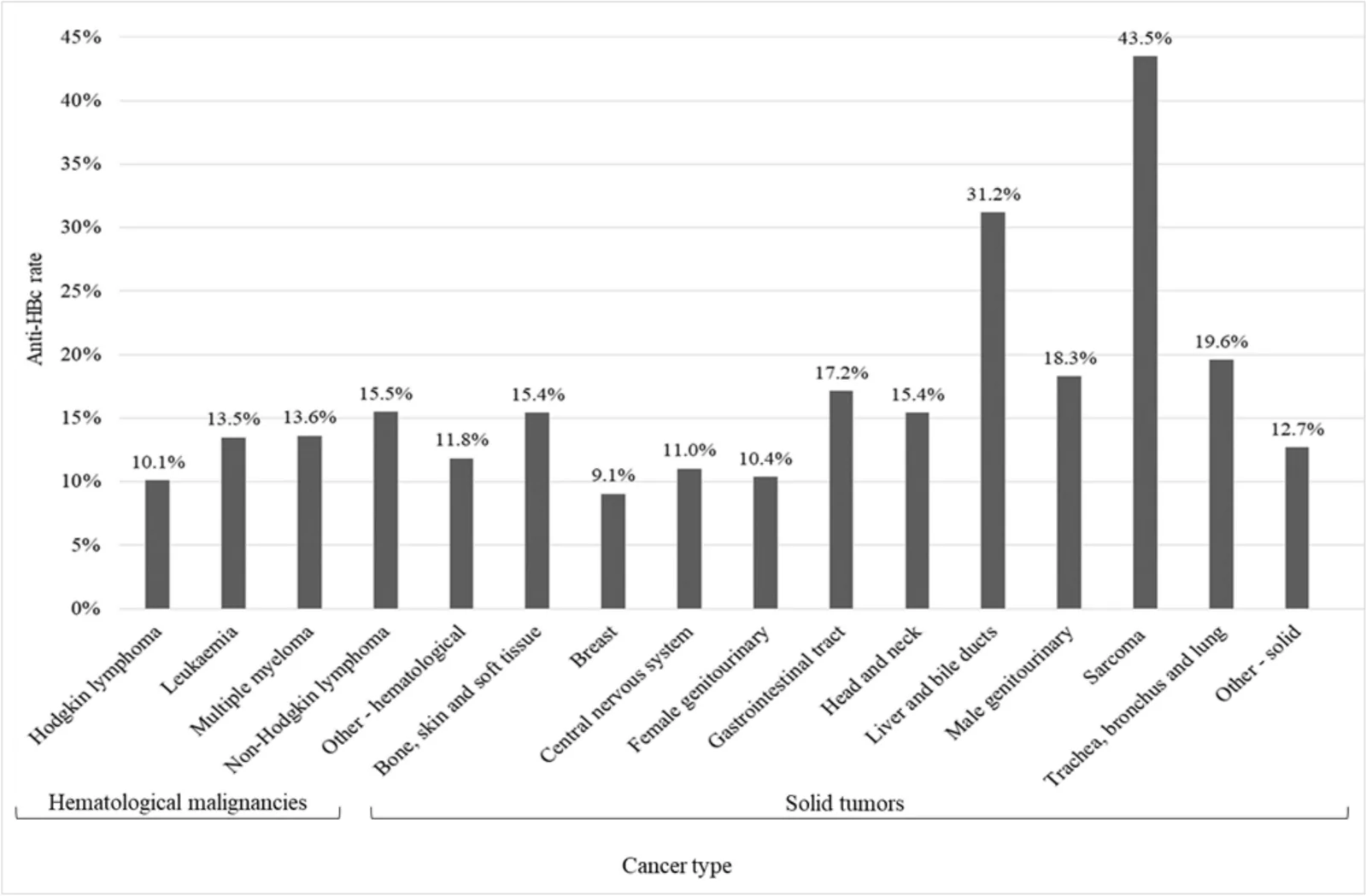
Seroprevalence of hepatitis B virus exposure among cancer patients by primary site of hematological malignancies and solid tumors, 2010–2023 *Anti-HBc* antibody against the hepatitis B core antigen

Among the study population, the seroprevalence of HBV exposure was 13.8% (885/6,406) in patients with hematological malignancies, 13.4% (2,752/20,571) in those with solid tumors, and 11.6% (220/1,892) among patients with missing data on cancer type. The distribution of HBV exposure across cancer subgroups is presented in Fig. 3. Among hematological malignancies, higher HBV seroprevalence rates were observed in patients with non-Hodgkin lymphoma (15.5%) and multiple myeloma (13.6%). Among solid tumors, the highest seroprevalence rates were observed in patients with sarcoma (43.5%) and liver and bile ducts cancers (31.2%).

### Subgroup analysis of liver/bile ducts and gastrointestinal cancers

A subgroup analysis was conducted to evaluate the association of HBV exposure with liver and bile duct cancers and gastrointestinal cancers. In the multivariate analysis, after adjusting for age, sex, and ethnicity/color, liver and bile duct cancers remained strongly associated with HBV exposure (adjusted OR = 3.30; 95% CI: 2.78–3.92; *p* < 0.001). Gastrointestinal cancers were also independently associated with HBV exposure (adjusted OR = 1.41; 95% CI: 1.26–1.58; *p* < 0.001) (Supplementary Table S2). The link for Supplementary Table S2 is not active.

### Factors associated with HBV exposure

We investigated whether the available variables were associated with the presence of HBV exposure. In univariate analysis, cancer type was not significantly associated with HBV exposure (*p* = 0.371) (Table 3). In contrast, age, sex, and ethnicity/color were independently associated with HBV exposure in multivariable analysis (*p* < 0.05). Patients aged ≥ 60 years had 4.4-fold higher odds of HBV exposure compared to those aged up to 39 years (18.1% *vs*. 4.7%; OR = 4.40; 95% CI: 3.77–5.12). Men had 89.0% higher odds of exposure compared to women (18.7% *vs*. 10.6%; OR = 1.89; 95% CI: 1.76–2.04). Additionally, non-White patients had 25.0% higher odds of exposure compared to White patients (16.8% *vs*. 14.8%; OR = 1.25; 95% CI: 1.15–1.35).

**Table 3:** Factors related to the presence of hepatitis B virus exposure among cancer patients, 2010–2023

Variables	Anti-HBc		OR* (95% CI) crude	p*	OR** (95% CI) adjusted	p**
	Negative n (%)	Positive *n* (%)				
Age, years						
18–39	4,362 (95.3)	216 (4.7)	1.00	–	1.00	–
40–59	10,059 (88.5)	1,304 (11.5)	2.62 (2.26–3.04)	–	2.79 (2.38–3.26)	< 0.001
≥ 60	10,591 (81.9)	2,337 (18.1)	4.46 (3.86–5.15)	< 0.001	4.40 (3.77–5.12)	< 0.001
Sex						
Female	16,895 (89.4)	1,993 (10.6)	1.00		1.00	
Male	8,117 (81.3)	1,864 (18.7)	1.95 (1.82–2.09)	< 0.001	1.89 (1.76–2.04)	< 0.001
Ethnicity/color						
White	15,097 (85.2)	2,624 (14.8)	1.00		1.00	
Non-White	4,623 (83.2)	933 (16.8)	1.16 (1.07–1.26)	< 0.001	1.25 (1.15–1.35)	< 0.001
Cancer type						
Hematological	5,521 (86.2)	885 (13.8)	1.00	–	–	–
Solid	17,819 (86.6)	2,752 (13.4)	0.96 (0.89–1.05)	0.371	–	–

*Anti-HBc* antibody against the hepatitis B core antigen, *CI* confidence interval, *OR* odds ratio

^*^Bivariate logistic regression

^**^Multivariate logistic regression

## Discussion

In this 14-year study conducted at a quaternary referral cancer center in Brazil, the overall seroprevalence of HBV exposure among cancer patients was 13.4% (3,857/28,869). This estimate is consistent with a previous Brazilian study reporting a prevalence of 13.9% (186/1,659) in patients with cervical cancer [13], although it is higher than that reported in some international cohorts, such as Ramsey et al., who found a prevalence of 6.5% (197/3,050) [14]. In contrast, studies from higher-endemicity settings such as Turkey have reported substantially higher prevalences among cancer patients (38.4%; 172/448) [15], thus highlighting the influence of regional epidemiological patterns on HBV prevalence.

The seroprevalence of HBV exposure among cancer patients in the present study appears to be higher than those reported for the general population. However, direct comparisons should be interpreted cautiously because most epidemiological studies in the general population are based only on HBsAg positivity, whereas HBV exposure in our study was defined by anti-HBc positivity. The global seroprevalence of HBsAg was recently estimated at approximately 2.9%, whereas in Latin America it was estimated at 1.0% [3, 16]. In Brazil, 302,351 new cases were reported between 2000 and 2024, most of them in the Southeastern region (34.0%; *n* = 102,721), including 63,670 cases in the state of São Paulo alone [17]. In Northeastern Brazil, HBV seroprevalence in the general population of an urban center was estimated at 0.24%, with most affected individuals self-identifying as Black/Brown [18]. Nevertheless, epidemiological studies incorporating anti-HBc positivity have also reported higher prevalence estimates. For example, a Brazilian review including anti-HBc and/or HBsAg positivity reported prevalence rates ranging from 8.2 to 11.1% among healthcare workers [19].

A key finding of this study was the significant decline in HBV seroprevalence among cancer patients over time (2010–2023), dropping from 16.5 to 9.9%. This trend is consistent with findings in the general population, including that in the United States, where HBV prevalence has steadily declined over recent decades [20]. This reduction may partly reflect the expansion of hepatitis B vaccination coverage. Additionally, evolving HBV screening practices may have contributed to the decline in anti-HBc positivity by increasing testing in lower-risk patients. However, data on testing indications or institutional screening policies were unavailable, precluding further evaluation. Evidence from countries such as China demonstrates the population-level impact of vaccination policies, with substantial reductions in chronic HBV infection between 1990 and 2020 [21]. In Brazil, the progressive expansion of universal vaccination likely contributed to these findings, particularly among younger individuals, including those with cancer [22]. However, individual vaccination data were not available for collection in the present study.

Declining trends were observed in both sexes, with a more pronounced reduction among men, which is consistent with findings from China [23] despite the higher proportion of HBV exposure in men. Multivariable analysis identified men, age ≥ 60 years, and non-White ethnicity/color as factors independently associated with HBV exposure. The higher seroprevalence among men has been previously reported in both general and cancer populations [11, 20, 24] and may be partially explained by biological and behavioral differences. Sexual dimorphism influences immune responses, with men exhibiting less effective responses to infectious agents. This is potentially related to the immunosuppressive effects of androgens, whereas estrogen may exert immunostimulatory effects, being associated with more robust vaccine responses in women [25].

A significant reduction in HBV seroprevalence was also observed across all age groups over time, including among older adults. Similar temporal trends were reported by Xu et al. between 2013 and 2020 [23]. Nevertheless, age ≥ 60 years remained independently associated with infection, likely reflecting cumulative lifetime exposure and reduced vaccine responsiveness. Globally, HBV prevalence increases with age [11, 26]. In a previous study, prevalence reached 9.3% among cancer patients older than 50 years compared to 4.3% among younger patients [11]. Reduced immunogenicity of hepatitis B vaccines in older adults may further contribute to increased susceptibility [27], in addition to lower vaccination coverage among cohorts not included in neonatal immunization programs [26, 28].

As for ethnicity/color, the temporal decline in HBV seroprevalence was significant among White patients, but not among non-White patients, who were also independently associated with HBV exposure. Higher HBV seroprevalence among non-White individuals has been consistently documented [13, 20, 29]. In a Brazilian study, Black men had 3.28-fold higher odds of anti-HBc positivity [29]. These findings highlight persistent inequities in social determinants of health, including access to vaccination, diagnosis, and treatment. Similarly, a large North American study involving 356,828 individuals demonstrated lower adherence to complete vaccination schedules among Black individuals [30].

With respect to cancer type, a significant decline in HBV seroprevalence was observed among patients with solid tumors, whereas no significant trend was identified among those with hematological malignancies. No association was found between cancer type and HBV exposure, which is consistent with previous reports [15]. In patients with solid tumors, therapies involving immune checkpoint inhibitors (e.g. nivolumab) may influence viral infection dynamics [31]. Conversely, patients with hematological malignancies are at increased risk of infection because of more profound immunosuppression [32]. Additionally, lower vaccine responsiveness [33, 34] and the frequent use of B-cell–depleting therapies (e.g. rituximab) further increase the risk of HBV reactivation [35].

(e.g. rituximab) further increase the risk of HBV reactivation [35].

When analysing subgroups of hematological malignancies, a higher prevalence was observed among patients with non-Hodgkin lymphoma (15.5%), a malignancy previously associated with HBV infection [36]. Evidence from meta-analyses suggests a possible relationship between HBV infection and the development of this malignancy, although the underlying mechanisms have not yet been fully elucidated [37]. Furthermore, prophylactic anti-HBV treatment has proven effective in preventing HBV reactivation in anti-HBc-positive/HBsAg-negative patients with T-cell lymphoma [38]. As for solid tumors, the high prevalence observed among patients with sarcomas (43.5%) should be cautiously interpreted, particularly given the small number of cases, which may have led to an overestimation of this prevalence.

An additional subgroup analysis demonstrated that liver and bile duct cancers and gastrointestinal cancers were independently associated with HBV exposure after adjustment for age, sex, and ethnicity/color. Patients with liver and bile duct cancers had more than threefold higher odds of HBV exposure, while those with gastrointestinal cancers had 41% higher odds. The strong association with liver and bile duct cancers is consistent with the well-established oncogenic role of HBV, which is mediated by persistent hepatic inflammation, viral DNA integration into the host genome, and the oncogenic effects of viral proteins such as HBx [1, 39]. Similarly, a recent meta-analysis reported that HBV infection was associated with an increased risk of biliary tract cancers (pooled OR = 2.16; 95% CI: 1.73–2.69) [40], while a large prospective cohort study found an increased risk of gastrointestinal cancers (HR = 5.59; 95% CI: 4.84–6.45), including colorectal, pancreatic, and gallbladder or extrahepatic bile duct cancers, among individuals with chronic HBV infection [41]. Although causality cannot be inferred from our findings, these results reinforce existing evidence linking HBV infection to hepatobiliary and gastrointestinal cancers.

Anti-HBc testing in cancer patients increased from 11.1% in 2010 to 29.2% in 2023, although a considerable proportion of admitted cancer patients remained untested. Despite the current recommendations for HBV screening in cancer patients, uptake remains suboptimal, particularly among those with solid tumors [11, 12]. A recent systematic review and meta-analysis showed that while global pre-chemotherapy screening rates rose from 37% (2006–2010) to 69% (2016–2020), the overall pooled rate was just 57%, underscoring persistent gaps in screening implementation [42]. This discrepancy may reflect a higher perceived risk associated with hematological malignancies, whose treatments generally involve greater immunosuppression [12]. In addition, chronic HBV infection has been linked to certain hematological malignancies, particularly non-Hodgkin lymphoma [37]. Nevertheless, cancer type was not independently associated with anti-HBc positivity in the present study, and testing remained limited. In this context, the implementation of systematic HBV screening should be strengthened in oncology care.

This study has limitations inherent to its retrospective design, including incomplete data within electronic medical records. Additionally, information on institutional HBV screening practices and the clinical indications for anti-HBc testing was unavailable. Consequently, anti-HBc testing may not have been performed systematically; furthermore, because inclusion was restricted to patients with documented HBV serological results, selection bias cannot be ruled out. Although additional HBV serological and molecular markers were available for a subset of anti-HBc-positive patients, these complementary data were not available for all individuals. Therefore, the primary analyses were based on anti-HBc positivity, which indicates prior exposure to HBV but does not, on its own, distinguish between resolved and current infection. Information on the timing of anti-HBc testing relative to cancer diagnosis and treatment was also unavailable. Treatment-related immunosuppression may have reduced antibody detectability, potentially leading to an underestimation of previous HBV exposure. Nevertheless, anti-HBc positivity remains highly relevant in oncology settings because it identifies patients at risk for HBV reactivation during immunosuppressive therapy. Finally, as a single-center study, the generalizability of our results may be limited. However, the large sample size and extended study period strengthen the robustness of these analyses. Furthermore, conducting this study at one of the largest oncology centers in Latin America enhances the epidemiological relevance of our findings.

## Conclusion

In conclusion, despite a significant decline in HBV seroprevalence over time, previous HBV exposure remains substantial among cancer patients, particularly among men, older adults, and non-White individuals. Persistent disparities across demographic subgroups reinforce the need for equitable prevention measures and systematic HBV screening in routine oncology care.

## Supplementary Information

Below is the link to the electronic supplementary material.

Supplementary file1 (DOCX 16 KB)

## Data Availability

The data that support the findings of this study are available from the corresponding author on reasonable request.
