# Supplementary material for: Trends in hepatitis B virus seroprevalence in a large cohort of adult cancer patients from 2010 to 2023
Source: Cancer Causes Control. 2026 Oct 7;37(10):178. doi: 10.1007/s10552-026-02262-w (PMC13646014; doi:10.1007/s10552-026-02262-w)
Supplement: Supplementary file 1 [file 10552_2026_2262_MOESM1_ESM.docx]

**Trends in hepatitis B virus seroprevalence in a large cohort of adult cancer patients from 2010 to 2023**

**Supplementary Table S1. Distribution of additional hepatitis B virus serological and molecular markers among anti-HBc-positive cancer patients**

| **HBV markers** | **Tested**  **n** | **Anti-HBc** | |
| --- | --- | --- | --- |
|  |  | **Negative**  **n (%)** | **Positive**  **n (%)** |
| HBsAg | 3,840 | 3,541 (92.2) | 299 (7.8) |
| Anti-HBs | 3,835 | 1,027 (26.8) | 2,808 (73.2) |
| Anti-HBc IgM^a^ | 564 | 543 (96.2) | 18 (3.2) |
| HBeAg | 558 | 500 (89.6) | 58 (10.4) |
| Anti-HBe | 284 | 75 (26.4) | 209 (73.6) |
| HBV DNA | 484 | 360 (74.4) | 124 (25.6) |

^a^ Inconclusive laboratory result: 3

*Anti-HBc* antibody against the hepatitis B core antigen, *HBV* hepatitis B virus

**Supplementary Table S2. Association of liver and bile duct and gastrointestinal cancers with hepatitis B virus exposure, 2010–2023**

| **Variables** | **Anti-HBc** | | | **OR* (95% CI)**  **crude** | **p*** | **OR** (95% CI)**  **adjusted** | **p**** |
| --- | --- | --- | --- | --- | --- | --- | --- |
|  | **Negative**  **n (%)** | **Positive**  **n (%)** | |  |  |  |  |
| **Liver/biliary cancer** | | |  |  |  |  |  |
| **No** | 22,787 (87.1) | 3,386 (12.9) | | 1.00 |  | 1.00 |  |
| **Yes** | 553 (68.8) | 251 (31.2) | | 3.06 (2.62-3.56) | < 0.001 | 3.30 (2.78-3.92) | < 0.001 |
| **Gastrointestinal cancer** | | |  |  |  |  |  |
| **No** | 20,626 (87.0) | 3,075 (13.0) | | 1.00 |  | 1.00 |  |
| **Yes** | 2,714 (82.8) | 562 (17.2) | | 1.39 (1.26-1.53) | < 0.001 | 1.41 (1.26-1.58) | < 0.001 |

* Bivariate logistic regression; ** Multivariate logistic regression adjusted for age, sex and ethnicity/color.

*Anti-HBc* antibody against the hepatitis B core antigen, *CI* confidence interval, *OR* odds ratio
